# Development and evaluation of an ultrasound-triggered microbubble combined transarterial chemoembolization (TACE) formulation on rabbit VX2 liver cancer model

**DOI:** 10.7150/thno.45348

**Published:** 2021-01-01

**Authors:** Doyeon Kim, Jae Hwan Lee, Hyungwon Moon, Minkyu Seo, Hyounkoo Han, Hongkeun Yoo, Howon Seo, Jingu Lee, Sujung Hong, Pilhan Kim, Hak Jong Lee, Jin Wook Chung, Hyuncheol Kim

**Affiliations:** 1 Department of Chemical & Biomolecular Engineering, Sogang University, 35 Baekbeom-ro, Mapo-gu, Seoul 04107, Republic of Korea.; 2 Department of Radiology, Seoul National University Bundang Hospital, 82, Gumi-ro 173beon-gil, Bundang-gu, Seongnam-si, Gyeonggi-do, Republic of Korea.; 3 IMGT Co., Ltd., 172 Dolma-ro, Bundang-gu, Seongnam 13605, Republic of Korea.; 4 Graduate School of Nanoscience and Technology, Korea Advanced Institute of Science and Technology (KAIST), Daejeon 34141, Republic of Korea.; 5 KI for Health Science and Technology (KIHST), Korea Advanced Institute of Science and Technology (KAIST), Daejeon, 34141, Republic of Korea.; 6 Graduate School of Medical Science and Engineering, Korea Advanced Institute of Science and Technology (KAIST), Daejeon, 34141, Republic of Korea.; 7 Department of Radiology, Seoul National University College of Medicine, Daehak-ro, Jongno-gu, Seoul, 03080, Republic of Korea.; 8 Institute of Radiation Medicine, Seoul National University Hospital, 101, Daehak-ro, Jongno-gu, Seoul, 03080, Republic of Korea.; 9 Department of Biomedical Engineering, Sogang University, 35 Baekbeom-ro, Mapo-gu, Seoul 04107, Republic of Korea.

**Keywords:** transarterial chemoembolization, ultrasound microbubble, sonoporation, theranostics, nanomedicine, hepatocellular carcinoma

## Abstract

Transarterial chemoembolization (TACE) is an image-guided locoregional therapy used for the treatment of patients with primary or secondary liver cancer. However, conventional TACE formulations are rapidly dissociated due to the instability of the emulsion, resulting in insufficient local drug concentrations in the target tumor.

**Methods:** To overcome these limitations, a doxorubicin-loaded albumin nanoparticle-conjugated microbubble complex in an iodized oil emulsion (DOX-NPs-MB complex in Lipiodol) has been developed as a new ultrasound-triggered TACE formulation.

**Results:** (1) Microbubbles enhanced therapeutic efficacy by effectively delivering doxorubicin- loaded nanoparticles into liver tumors via sonoporation under ultrasound irradiation (US+). (2) Microbubbles constituting the complex retained their function as an ultrasound contrast agent in Lipiodol. In a rabbit VX2 liver cancer model, the *in vivo* study of DOX-NPs-MB complex in Lipiodol (US+) decreased the viability of tumor more than the conventional TACE formulation, and in particular, effectively killed cancer cells in the tumor periphery.

**Conclusion:** Incorporation of doxorubicin-loaded microbubble in the TACE formulation facilitated drug delivery to the tumor with real-time monitoring and enhanced the therapeutic efficacy of TACE. Thus, the enhanced TACE formulation may represent a new treatment strategy against liver cancer.

## Introduction

Image-guided intra-arterial therapies, such as transarterial chemoembolization (TACE), are valuable tools for the treatment of primary or secondary liver cancer 1, 2. The goal of TACE is to selectively deliver the anticancer agent-iodized oil emulsion (Lipiodol; Guerbet, Aulnay-sous-Bois, France) to the tumor arteries. Infusion of emulsion results in targeted delivery of high concentrations of anticancer drug directly to the tumor, and blocks the terminal arteriole using Lipiodol 3, 4. TACE has been adopted as a standard treatment option for intermediate-stage hepatocellular carcinoma (HCC) 5. However, clinical outcomes with TACE are still unsatisfactory and typically elicit only a partial response in 15-55% of patients, and increase the median survival from 16 to 20 months 6, 7. The poor outcome may be partly explained by the low efficiency of drug delivery to tumors 8. During the TACE procedure, a considerable proportion of the chemotherapeutic agent enters the systemic blood circulation due to instability of the emulsion, resulting in low drug delivery efficiency and systemic side effects. It has been postulated that insufficient local delivery of chemotherapeutic agents with arterial embolization, induces tumor hypoxia, resulting in neo-angiogenesis and metastasis, and is subsequently associated with poor outcomes 9, 10. Adverse drug reactions, such as fatigue, weight loss, pain and nausea, induced by anticancer drugs leaking into the systemic circulation are another clinical challenge. Thus, the establishment of a new TACE drug delivery system, which facilitates drug delivery and real-time imaging, is essential for enhanced tumor control and reduced side effects.

**Figure 1:**
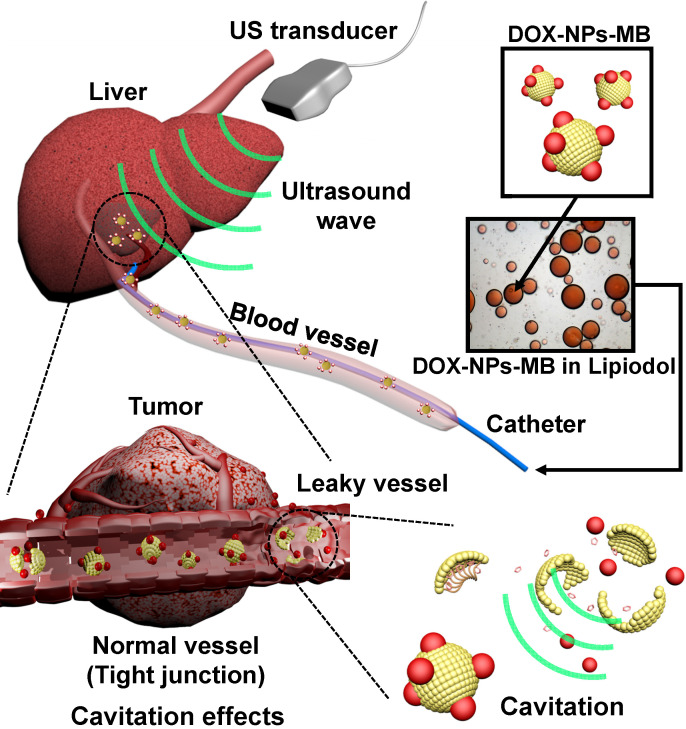
Schematic illustration showing the use of newly developed DOX-NPs-MB complex in Lipiodol formulation to enhance drug delivery via ultrasound irradiation (US+) during TACE procedure.

To overcome the limitations of current TACE formulations, an aqueous phase of ultrasound-triggered, doxorubicin-loaded, nanoparticle-conjugated microbubble complexes was emulsified in Lipiodol (DOX-NPs-MB complex in Lipiodol) (Figure 1). In the last decade, microbubbles have been actively studied not only as ultrasound contrast agents but also for local drug delivery under ultrasound exposure (US+) 11, 12. Theoretically, microbubbles exposed to ultrasound waves experience repeated volumetric expansion and contraction 13, 14. The oscillation propagates microstreams in blood vessels, and epithelial junctions near the microbubble become leaky. In addition, following a collapse under strong ultrasonic exposure, the microbubble generates microjets and a shock wave. The microjet and shock wave temporarily induce pores in the cell membrane, and renders the membrane permeable to drug nanoparticles (sonoporation effect) 15-17. Thus, when the DOX-NPs-MB complex in Lipiodol is infused intra-arterially, DOX-NPs-MB complex is localized near the blood vessel wall and the cavitation of microbubbles under the exposure of ultrasound generates jet streams and shock waves, which weakens the hepatic tumor vessel wall. Consequently, the chemotherapeutic agent encapsulating nanoparticles, which are conjugated on the surface of microbubbles, are delivered deep inside the tumor through the weakened blood vessel wall with the help of the jet stream (Figure 1) 18. Thus, the cavitation of microbubbles under ultrasound treatment improves drug delivery. Simultaneously, the TACE procedure can be monitored in real time using ultrasound.

## Methods

### Materials

Reagents including 1,2-Distearoyl-sn-glycero-3-phosphocholine (DSPC) and 1,2-distearoyl-*sn*-glycero-3-phosphoethanolamine-N-[amino(polyethylene glycol)2000] (Ammonium Salt) (DSPE-PEG2k-NHS) were purchased from Avanti Polar Lipids, Inc. (Alabaster, USA). Human serum albumin (HSA), 2-iminothiolane hydrochloride, and 99% ethanol were acquired from Sigma-Aldrich (St. Louis, MO, USA). Doxorubicin hydrochloride was supplied by Il-Dong Pharmaceutical Company (Seoul, Korea) in a dry powder form. Microcatheters (Progreat 2.0-F, Terumo, Japan) were obtained from the Terumo Korea Corporation (Seoul, Korea). Rabbits were ordered from Orient Bio Co. (Seongnam, Korea). Lipiodol was procured from Gurbet (Aulnay-sous-Bois, France). All other chemicals and solvents were prepared as analytical grade.

### Synthesis of DOX-NPs

The doxorubicin-loaded human serum albumin nanoparticles were fabricated by desolvation method 19. Albumin was dissolved in distilled water at a concentration of 40 mg/mL and the pH was adjusted to 8-8.5 with 0.2 M NaOH. Thiolation was performed with 2-iminothiolane hydrochloride (2-IT, 7.5 mg/mL) and the solution was reacted in a 25 °C shaker for 1 h. The solution was centrifuged twice at 4,000 rpm for 15 min using Amicon Ultra-30 KDa Centricons, in order to remove unreacted 2-IT after thiolation of albumin. Next, doxorubicin-HCl (10 mg/mL) was added to the albumin solution. The nanoparticle was obtained by the dropwise addition of ethanol to the mixture of doxorubicin and albumin at a rate of 1 mL/min until the solution became turbid (desolvation) following stirring. The reaction mixture was stirred gently for 1 day. The solution was centrifuged at 13,200 rpm for 10 min and the supernatants were collected to confirm the encapsulation efficiency of doxorubicin. The doxorubicin-loaded albumin nanoparticles were dispersed in a contrast agent (Pamiray 250, Dongguk Pharmaceutical, Seoul, Korea). The encapsulation efficiency of doxorubicin was calculated via high-performance liquid chromatography (HPLC) of unbound doxorubicin in the supernatants after centrifugation, based on the following equation.


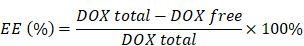


### Synthesis of DOX-NPs-MB complex

Microbubbles were created by dissolving DSPE-PEG2k-NHS and DSPC in chloroform at a molar ratio of 1:9. The chloroform was fully evaporated to obtain a thin phospholipid film. The phospholipid film was hydrated with 1 mg/mL PBS at pH 7.4. The dispersed solution in the vial was filled with C_3_F_8_ gas. Microbubbles were formed by vigorous agitation with a Vialmix^TM^ for 45 s. Microbubbles were transferred to a 5 mL syringe and centrifuged at 1,500 rpm for 15 min to induce floatation. The lower portion without microbubbles was discarded and the upper portion containing the microbubbles was dispersed in a DOX-NPs solution to form the DOX-NPs-MB complex. The albumin-doxorubicin nanoparticles were conjugated to the microbubbles via amide bonds between the primary amine of the nanoparticles and the NHS from the microbubble surface. The mixture of DOX-NPs and MB solution was reacted for 1 h 30 min or more at room temperature.

### Preparation of DOX-NPs-MB complex in Lipiodol for chemoembolization

The DOX-NPs-MB complex containing a doxorubicin concentration of 6.25mg/mL (aqueous phase) was dispersed in a Lipodol (oil phase) to form the DOX-NPs-MB complex in Lipiodol. An emulsion was obtained by mixing the aqueous and oil phases at a ratio of 1:2 using a Voltexer machine at least 5 times for 5 s. The DOX in Lipiodol group (conventional TACE formulation) was generated using a 3-way pumping method.

### *In vitro* release of doxorubicin from the DOX-NPs-MB complex in Lipiodol

The rate of *in vitro* release of DOX was determined using two formulations: DOX in Lipiodol and DOX-NPs-MB complex in Lipiodol with or without ultrasound irradiation. Each group was loaded into the dialysis membrane (Spectra/Por^®^7, MWCO: 2 kD) and transferred into a tube filled with 10 mL PBS. Dialysis membranes were maintained at a constant temperature of 37 °C in a shaking incubator at 500 rpm. The doxorubicin release was measured via HPLC. The mobile phase consisted of acetonitrile and distilled water in the ratio of 50: 50 with phosphoric acid 0.6 mL/L and sodium dodecyl sulfate 1.327 g/L.

### Ultrasound imaging of the emulsion containing DOX-NPs-MB complex in Lipiodol

Conventional ultrasound imaging equipment (IU-22, Philips Medical System, Philips, Bothell, WA, USA) was used to determine whether the microbubbles in DOX-NPs-MB complex act as a contrast agent (mechanical index: 0.06). Ultrasonic shocks were also applied four times via flash buttons installed in the instrument (Mechanical index: 0.15) to confirm the cavitation of MB under ultrasound irradiation.

### Morphology of the emulsion formulation containing DOX-NPs-MB complex in Lipiodol following intravascular infusion

The size of DOX-NPs-MB complex in Lipiodol emulsion was larger than the diameter of capillaries in the tumor. Hence, a microfluidic channel was fabricated to determine the behavior of intra-arterially infused DOX-NPs-MB complex in Lipiodol in the peripheral capillaries of the tumor. First, the microfluidic channels mimicking blood capillary vessels were fabricated by: (1) designing the microfluidic channel map using CAD software, (2) fabricating a semiconductor-based photomask (Nepco in Gyeonggi-do, Korea) with the CAD map; (3) making silicon wafers on which a microfluidic channel map was installed (request Amed in Seoul, Korea); (4) pouring the PDMS solution (Polydimethylsiloxane:Curing Agent = 10:1) on the surface of silicon wafer and the glass; (5) incubating the silicon wafer in an oven at 120 °C for 12 h; (6) attaching the -OH functional group onto the surface of the two molds; and (7) attaching the two molds via a dehydration condensation reaction. The emulsion formulation of DOX-NPs-MB complex in Lipiodol was infused into the microfluidic channel using a 1 mL syringe. The fluorescence of DOX-NPs was imaged to measure the quantity of DOX-NPs-MB complex in Lipiodol formulation in the narrow microfluidic channels.

### Visualization of intra-arterially infused emulsion of DOX-NPs-MB complex in Lipiodol using an IVMV (*in vivo* micro-visualization) imaging system

To visualize liver tissues obtained from rabbit VX2 liver tumors, a custom-built video-rate confocal laser-scanning microscope was used 20-22. Three continuous-wave laser modules with output wavelengths of 488 nm (MLD488, Cobolt), 561 nm (Jive, Cobolt), and 640 nm (MLD640, Cobolt) were used as excitation light sources. Two-dimensional laser scans were achieved using a rapidly rotating polygonal mirror with 36 facets (MC-5, Lincoln Laser) for fast-axis scanning, and a galvanometer scanning mirror (6230H, Cambridge Technology) for y-axis scanning. The multi-color fluorescence signals were captured by a high NA commercial objective lens (CFI Plan Apo λ, 10X, NA0.45, Nikon; UPLSAP0, 4X, NA 0.16, Olympus), and detected by three photomultiplier tubes (PMT; R9110, Hamamatsu). The output signals of each PMT were digitally acquired by frame grabber (Solios, Matrox), and the images were recorded and displayed with custom-written imaging software based on the Matrox Imaging Library (MIL9, Matrox) and Visual C++ 23.

### Tissue optical clearing of liver tumor samples

Samples fixed in 4% PFA (PFA; BPP-9016, T&I, diluted in PBS) were washed with phosphate-buffered saline (PBS; LB004-02, Welgene) for 1 min. The samples were sliced into 300-500-μm-thick sections and dehydrated with 80% (wt/vol) ethanol (EtOH CAS 64-17-5; 4022-4100, Daejung) for 1 day at room temperature (RT) in a shaker. For optical clearing, dehydrated samples were immersed in peroxide-free BABB solution for 1 day at RT in a shaker. Peroxide-free BABB solution was prepared by mixing 40 mL of 1:2 volume ratio of benzyl alcohol (402834, Sigma) and benzyl benzoate (B6630-1L, Sigma) with 10 g of aluminum oxide (Al_2_O_3_; 199443, Sigma). The supernatant was discarded after centrifuging the mixed solvents.

### Preparation of preclinical liver tumor model

Animal research protocols followed in this study were approved by Seoul National University of Medicine institutional animal care and use committee. Male New Zealand white rabbits (n = 35) weighing 3.0-3.5 kg were used. The animals were housed in cages under a 12-h light/dark cycle, with *ad libitum* access to standard rabbit chow diet and water. During all procedures, the animals were anesthetized with intramuscular injections of 5 mg/kg body weight of tiletamine-zolazepam (Zoletil 50, Virbac, Carros, France) and a 2 mg/kg body weight of 2% xylazine hydrochloride (Rompun; Bayer, Seoul, Korea). The VX2 carcinoma strain was maintained in the right hind limb of a carrier rabbit via deep intramuscular injection throughout the study. Briefly, the left lobe of the animal's liver was exposed surgically and a small piece of tumor tissue (1 mm^3^) freshly harvested from the tumor was directly implanted into the subcapsular area of the liver for each rabbit, as described in previous studies 24. The tumor was incubated for 17-18 days before treatment.

### MR imaging

All animals underwent MR imaging on day 0 (baseline before treatment), and on day 7 following TACE procedures in the treated groups and untreated controls. A 3.0-T clinical MR scanner (TimTrio, Siemens Healthcare, Erlangen, Germany) with a knee coil was used to improve SNR and spatial resolution. The animals were fixed on a board in a supine position, and an abdominal bandage was tightly applied to reduce any movement artifact. Axial T2-weighted turbo spin-echo (repetition time/echo time: 4,100 ms/150 ms; echo train length: 14; section thickness: 3 mm; field of view: 130 mm × 130 mm; matrix: 512 × 358; number of excitations: 2.0) and IVIM diffusion-weighted imagery (free-breathing single-shot echo-planar imaging pulse sequence with diffusion gradients applied in three orthogonal directions: 2,700/63; section thickness: 3 mm; number of sections: 20; number of signals acquired: 8; field of view: (14 cm × 14 cm); matrix: (128 × 128); and four b values of (0, 15, 200, and 800) s/mm^2^) was acquired 24. The images were evaluated using a dedicated picture archiving and communication system (PACS) workstation (m-view; Marotech, Seoul, Korea).

### Animal grouping and drug delivery

The animals were randomly divided into 4 groups with similar tumor volumes: animals treated with an intra-arterial (IA) infusion of doxorubicin solution in Lipiodol (DOX in Lipiodol group, n = 7)), doxorubicin-encapsulating nanoparticle-microbubble complex in Lipiodol with ultrasound irradiation (DOX-NPs-MB complex in Lipiodol (US+), n = 7), doxorubicin encapsulating nanoparticles-microbubble complex in Lipiodol without ultrasound irradiation (DOX-NPs-MB complex in Lipiodol (US-), n = 7), and untreated control (n = 6). The dose of doxorubicin delivered to the liver tumor tissue was 0.5 mg in all groups except for the control. An 18-gauge catheter (BD Angiocath Plus with intravenous catheter, Becton-Dickinson, Korea) was inserted into the right central auricular artery for arterial access, during the IA delivery. To access the correct hepatic artery, a 2.0-Fr microcatheter was advanced via the catheter into the descending aorta. Hepatic arteriography was performed to confirm tumor staining, and following visualization of the correct hepatic artery, the microcatheter was advanced selectively, until the catheter tip was gently positioned at the proximal portion of the proper hepatic artery. The mixture prepared for each group was then administered gently under fluoroscopic guidance. When the mixture was completely injected, the microcatheter was removed, and the puncture site was carefully compressed to achieve hemostasis.

### Ultrasound and microbubble activation

Abdominal hair was carefully removed immediately before ultrasonography. Ultrasonography was performed by a radiologist both before and during drug administration in the DOX-NPs-MB complex in Lipiodol group using the Aplio 500 ultrasonographic system (Toshiba Medical Systems, Otawara, Japan), equipped with 375BT Convex Probe Transducer with a center frequency of 3.5 MHz. A fundamental B-mode ultrasound (dynamic range of 65; mechanical index of 1.5; gain of 90; and depth of 4 cm) was used to detect the VX2 tumors. The optimal plane was determined after localization and morphological examination of the tumor, and the skin was marked. The vascular recognition imaging mode with a low MI of 0.06 was used to detect signals generated by the microbubbles. The vascular recognition mode was used to confirm tumor enhancement as soon as the IA delivery of the mixture was initiated. Simultaneously, the B-mode was used to irradiate ultrasound energy to the tumor for 15 min using a continuous up-and-down sweeping of the probe at the marked skin site.

### Imaging analysis

A radiologist, who was blinded to the experimental groups, evaluated the MR images on a PACS workstation. The T_2_-weighted images were used to confirm tumor formation and to measure the maximal longitudinal diameter (length) and maximal transverse diameter (width) of the tumors. Tumor volume was calculated based on MR imaging measurements, using the modified ellipsoidal formula, tumor volume = ½ (length × width^2^) 25, 26. The volume inhibition rate (VIR) of tumor growth was calculated using the formula, IR = (T_c_ - T_t_)/T_c_ × 100 %, where T_c_ represents the tumor volume of the control group, and T_t_ represents the tumor volume of each treatment group. The value of the apparent diffusion coefficient (ADC) was measured quantitatively using the largest cross-section of the tumor visualized on the ADC map. The changes in the ADC values before and after TACE were evaluated.

### Pathological analysis and liver toxicity assessment

All the animals were pre-anesthetized and sacrificed by administering an intravenous injection of xylazine hydrochloride on day 7, and the whole tumor was harvested after follow-up imaging. Each tumor was fixed in 10% buffered formalin. The specimen was then embedded in paraffin and cut into 4-µm-thick sections. The largest cross-section of the tumor was stained with hematoxylin and eosin for basic histopathological examination. The section was consecutively treated with terminal deoxynucleotidyl transferase dUTP nick-end labeling (TUNEL) staining (ApopTagfi peroxidase *in situ* apoptosis detection kit, Merck KGaA, Darmstadt, Germany) to evaluate tumor viability. The digital images of the histology slides were obtained (Leica Microsystems, Mannheim, Germany). The viable tumor percentage per tumor was calculated using image analysis software (ImageJ, version 1.45 s; National Institutes of Health, Bethesda, MD, USA). In brief, the viable portion of each TUNEL-stained image of the whole tumor was measured according to threshold intensity. The percentage of viable tumor region was calculated according to the ratio between the whole tumor area and the viable tumor region. This analysis was performed by an experienced radiologist who was blind to all the experimental data, in order to ensure concordance. The estimated viable tumor volume after treatment was calculated as follows: calculated tumor volume on day 7 multiplied by viable percentage of the tumor.

Blood samples for assessing liver toxicity were collected at baseline, and again on days 1, 3, and 7 after treatment. Liver function tests included the assessment of liver enzymes, aspartate transaminase (AST) and alanine transaminase (ALT).

### High-content screening (HCS) visualization of the tumor penetration of DOX-NPs

The emulsion containing Alexa 647-NHS dye-conjugated nanoparticles was injected into the VX2 tumor via the common hepatic artery to determine the penetration efficacy of DOX-NPs into tumor tissue with or without ultrasound irradiation in the group infused with DOX-NPs-MB complex in Lipiodol formulation. Cryo-sectioned slides were prepared with tumor tissue (fixed with 4% PFA (PFA; BPP-9016, T&I, diluted in PBS). Fluorescent images of the slide samples were obtained using the Operetta CLS High-Content Analysis system (Operetta CLS, PerkinElmer, Germany).

### Statistical analysis

All the study data are reported as means ± SD. Nonparametric analysis was conducted using the Kruskal-Wallis test to compare tumor volumes, volume inhibition rate, changes in ADC value, tumor viability, and estimated viable tumor volume in the four experimental groups. Positive results were subjected to Mann-Whitney *post hoc* test for one-to-one group comparison. Data processing and analysis were performed using the Statistical Package for the Social Sciences version 18.0 (SPSS Inc, IBM, Chicago, IL, USA). A two-sided p-value of less than 0.05 was considered statistically significant difference between the groups.

## Results

### Characterization of the emulsion containing DOX-NPs-MB complex in Lipiodol

**Figure 2:**
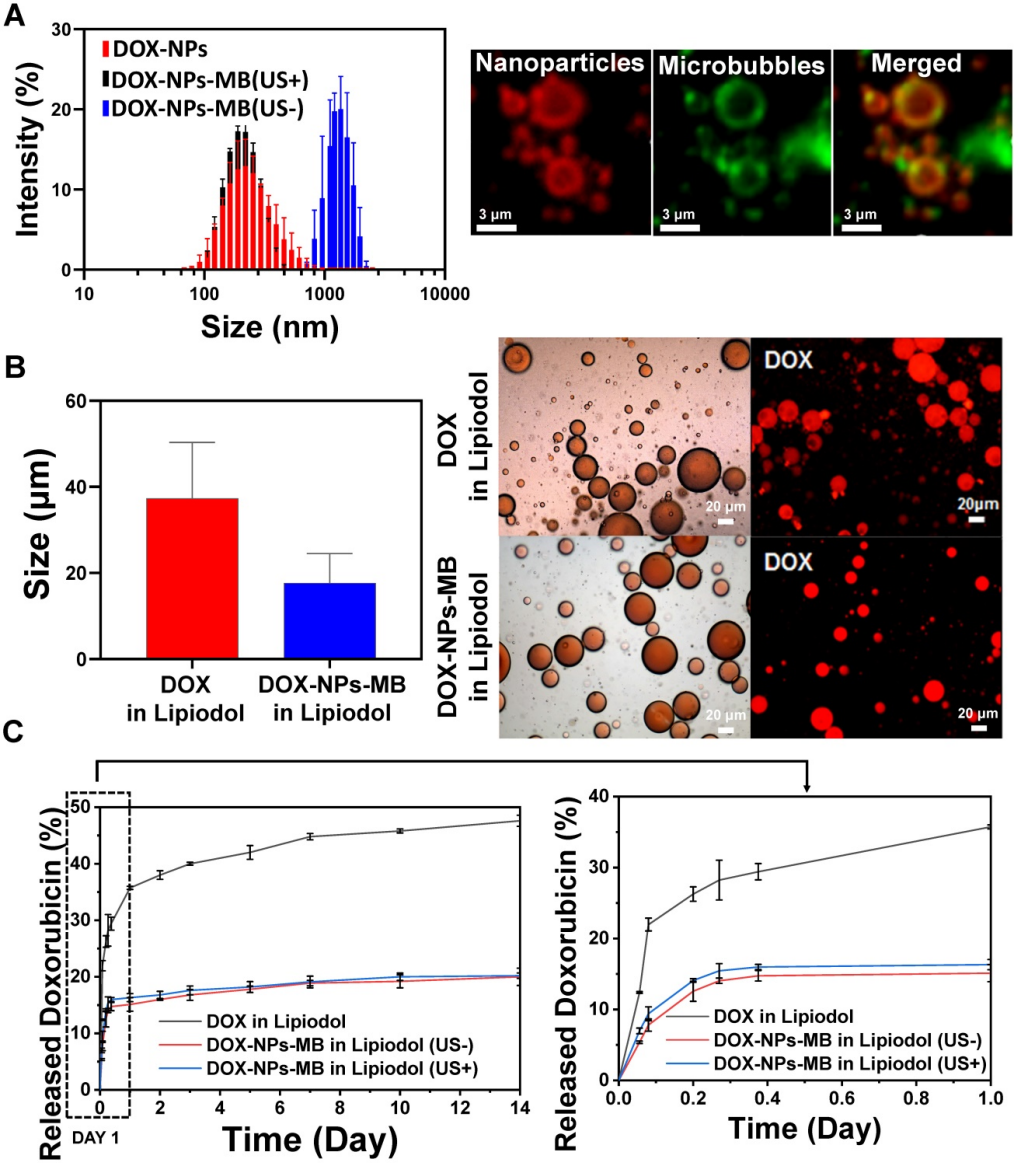
** Characterization of the emulsion containing DOX-NPs-MB complex in Lipiodol.** (A) Size distribution of the DOX-NPs and DOX-NPs-MB complex with (US+) and without (US-) ultrasound, and fluorescent images demonstrating the successful adsorption of DOX-NPs onto the surface of MB. Red fluorescence indicates DOX-NPs and green fluorescence indicates microbubbles. (B) Size analysis of emulsion droplets in the formulation of DOX in Lipiodol and DOX-NPs-MB complex in Lipiodol (left). DIC and fluorescent images demonstrate the emulsion droplet morphology of DOX in Lipiodol and DOX-NPs-MB in Lipiodol formulations (right). (C) *In vitro* release rate of doxorubicin in the following three groups: DOX in Lipiodol, DOX-NPs-MB complex in Lipiodol without ultrasound irradiation (US-), and DOX-NPs-MB complex in Lipiodol with ultrasound irradiation (US+). The graph presents doxorubicin release profile of each group for 14 days (left) and 1 day (right).

DOX-NPs-MB complex was prepared in first step to obtain the emulsion of DOX-NPs-MB complex in Lipiodol. The DOX-NPs showed a uniform size distribution, 198.30 ± 5.19 nm (Figure 2A, red color). The doxorubicin encapsulation efficiency of DOX-NPs was 67.2 ± 8.5%. The size of the DOX-NPs-MB complex was 1.24 ± 0.17 μm (Figure 2A, blue color). We analyzed whether the cavitation effect of ultrasound on DOX-NPs-MB complex was effective. The size of the DOX-NPs-MB complex after exposure to the ultrasound irradiation decreased to 225.0 ± 3.1 nm (Figure 2A, black), almost the same size as DOX-NPs, because of the effective disruption of MB. The fluorescence image as shown in Figure 2A demonstrates the successful conjugation of DOX-NPs onto the surface of MB via co-localization of DOX-NPs (red) and MB (green). The DOX-NPs-MB complex in contrast media (Pamiray 250, water phase) was emulsified in Lipiodol to develop a new TACE formulation. The emulsion size of DOX in Lipiodol, a conventional formulation used in the clinic (cTACE), was 37.3 ± 12.7μm (Figure 2B, red). In comparison, the size of emulsion of DOX-NPs-MB complex in Lipiodol was 17.7± 6.8 μm, which was relatively small compared with that of DOX in Lipiodol formulation. DIC images of DOX in Lipiodol and DOX-NPs-MB complex in Lipiodol exhibited uniform and stable emulsion morphology (Figure 2B, left images). As shown in Figure 2B, fluorescence images revealed that both DOX in Lipiodol and DOX-NPs-MB complex in Lipiodol contained the doxorubicin chemotherapeutic agent (red) in emulsion droplets. In addition, the DIC images of DOX-NPs-MB complex in Lipiodol emulsion formulation were obtained at different time points (Figure S1), demonstrating that the emulsion of DOX-NPs-MB complex in Lipiodol was stable for more than an hour.

### *In vitro* release of doxorubicin from the DOX-NPs-MB complex in Lipiodol formulation depending on ultrasound exposure

The initial doxorubicin concentration in DOX-NPs-MB complex aqueous solution was adjusted to 6.25 mg/mL. Following the emulsification of the aqueous complex in Lipiodol, the doxorubicin concentration was 2.08 mg/mL (aqueous solution:Lipiodol, 1:2). *In vitro* release of doxorubicin from the three groups was as follows: DOX in Lipiodol, DOX-NPs-MB complex in Lipiodol without ultrasound (US-), and DOX-NPs-MB complex in Lipiodol with ultrasound (US+) (Figure 2C). In the first 24 h, 35.73 ± 0.28% of encapsulated doxorubicin was released from the DOX in Lipiodol formulation, whereas 15.11 ± 1.2% was released within the same period from the DOX-NPs-MB complex in Lipiodol formulation without ultrasound (US-), and 16.32 ± 0.72% was released with ultrasound (US+). A slight increase in doxorubicin release occurred due to microbubble cavitation following US exposure. However, ultrasound had no effect on the sustained release of doxorubicin from the albumin nanoparticles.

### Ultrasonic response of DOX-NPs-MB complex in Lipiodol emulsion formulation

**Figure 3:**
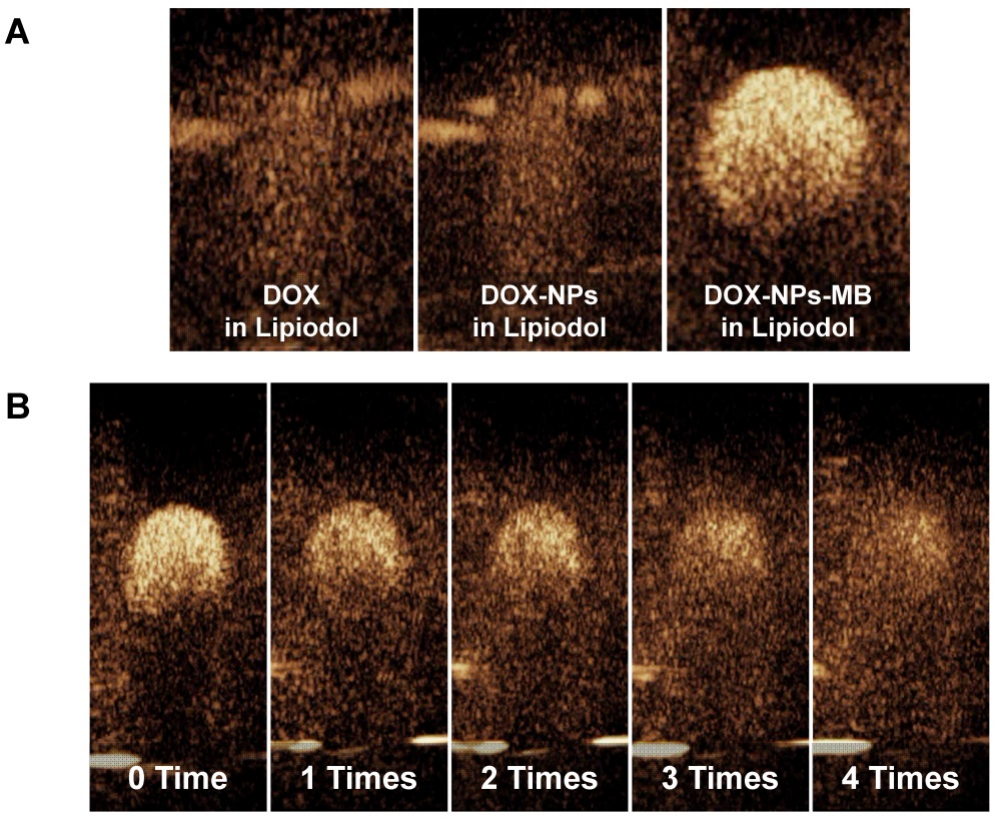
** Ultrasound contrast intensity of DOX-NPs-MB complex in Lipiodol emulsion formulation** (A) Ultrasound echogenicity of three types of formulations: DOX in Lipiodol, DOX-NPs in Lipiodol, and DOX-NPs-MB complex in Lipiodol; (B) Loss of echogenicity of DOX-NPs-MB complex in Lipiodol formulation due to microbubble cavitation with increasing frequency of ultrasound irradiations. Ultrasonic shocks were also applied four times via flash buttons installed in the instrument (Mechanical index: 0.15) to confirm the cavitation of MB under ultrasound irradiation.

The echogenicity of DOX-NPs-MB complex in Lipiodol emulsion was evaluated to determine whether microbubbles in the emulsion formulation resonated in response to ultrasound irradiation in real-time ultrasound imaging. The three formulations (DOX in Lipiodol, DOX-NPs in Lipiodol and DOX-NPs-MB in Lipiodol) were deposited inside the holes of a 2% agarose phantom. At a low MI of 0.06, the DOX-NPs-MB complex in Lipiodol was stably visualized (Figure 3A). However, no echogenicity was found in the other groups that did not carry microbubbles (DOX in Lipiodol, DOX-NPs in Lipiodol).

The effect of repeated ultrasound irradiation on echogenicity was investigated to determine the cavitation of microbubbles under ultrasound exposure (Figure 3B). Depending on the frequency of ultrasound irradiation, the echogenicity of microbubbles diminished as a result of microbubble cavitation. Although the delivery vehicle showed morphological features of emulsion, it can be monitored in real time via visualization of microbubbles. In addition, selective cavitation of microbubbles can be expected to improve the efficiency of drug delivery due to the sonoporation effect.

### Intravascular distribution of the DOX-NPs-MB complex in Lipiodol emulsion formulation

**Figure 4:**
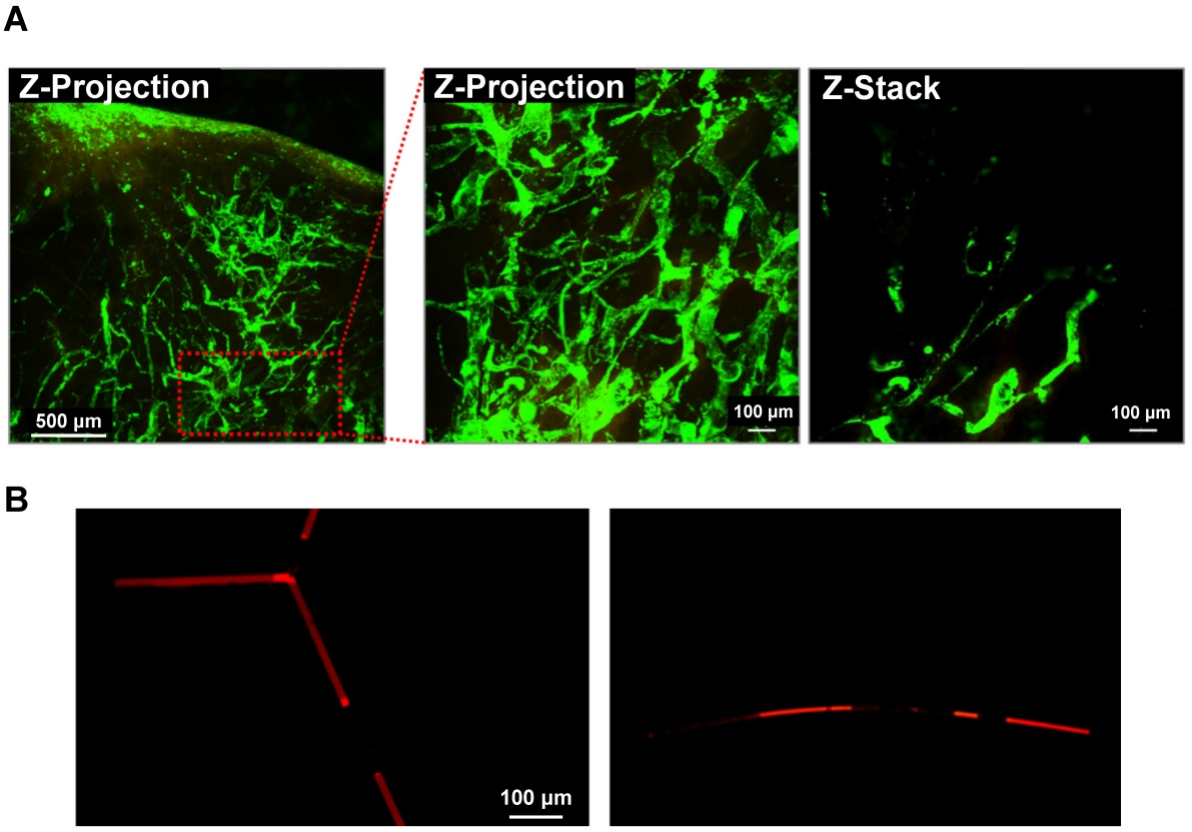
** Intravascular distribution of the emulsion of DOX-NPs-MB complex in Lipiodol** (A) Visualization of the emulsion of DOX-NPs-MB complex in Lipiodol in VX2 liver tumor using a custom-built video-rate laser-scanning confocal microscope (IVMV imaging system). Green fluorescence indicates the aqueous layer of DOX-NPs-MB complex. The scale bars indicate 500, 100 and 100 μm, respectively. (B) Layers of DOX-NPs-MB complex in Lipiodol in the blood vessel-like microfluidic system. Red fluorescence indicates the aqueous layer of DOX-NPs-MB complex. The scale bar indicates 100 μm.

When TACE was performed using the DOX-NPs-MB complex in Lipiodol emulsion, a custom-built video-rate laser-scanning confocal microscope (IVMV imaging system) was used to determine whether the formulation was infused into the hepatic blood vessels correctly. Figure 4A (including supplementary movie) demonstrates the visualization of the DOX-NPs-MB complex in Lipiodol emulsion in the hepatic blood vessels, demonstrating the infusion of the emulsion not only into the large blood vessels, but also into the micro-vessels on the surface of the liver tumor during *in vivo* studies. The size of the DOX-NPs-MB complex in Lipiodol emulsion was approximately 17 μm (Figure 2B). When the emulsion droplet was larger than the diameter of the blood vessels, it is important to evaluate the behavior of the emulsion in the blood vessel. Fluorescent doxorubicin encapsulated in DOX-NPs-MB complex was observed after the infusion of the DOX-NPs-MB complex in Lipiodol emulsion into the blood vessel-like microfluidic system, suggesting layers of aqueous phase containing DOX-NPs-MB complex and Lipiodol (Figure 4B). Thus, the DOX-NPs-MB complex in aqueous phase is in direct contact with the wall of the blood vessel, rather than an emulsion in the blood microvessels. Therefore, doxorubicin-encapsulating nanoparticles (DOX-NPs) might effectively penetrate the vessel wall and be delivered into the liver tumor upon cavitation of microbubbles following ultrasound treatment.

### Evaluation of pathology and anticancer efficacy of DOX-NPs-MB complex in Lipiodol via quantitative MR imaging

**Figure 5:**
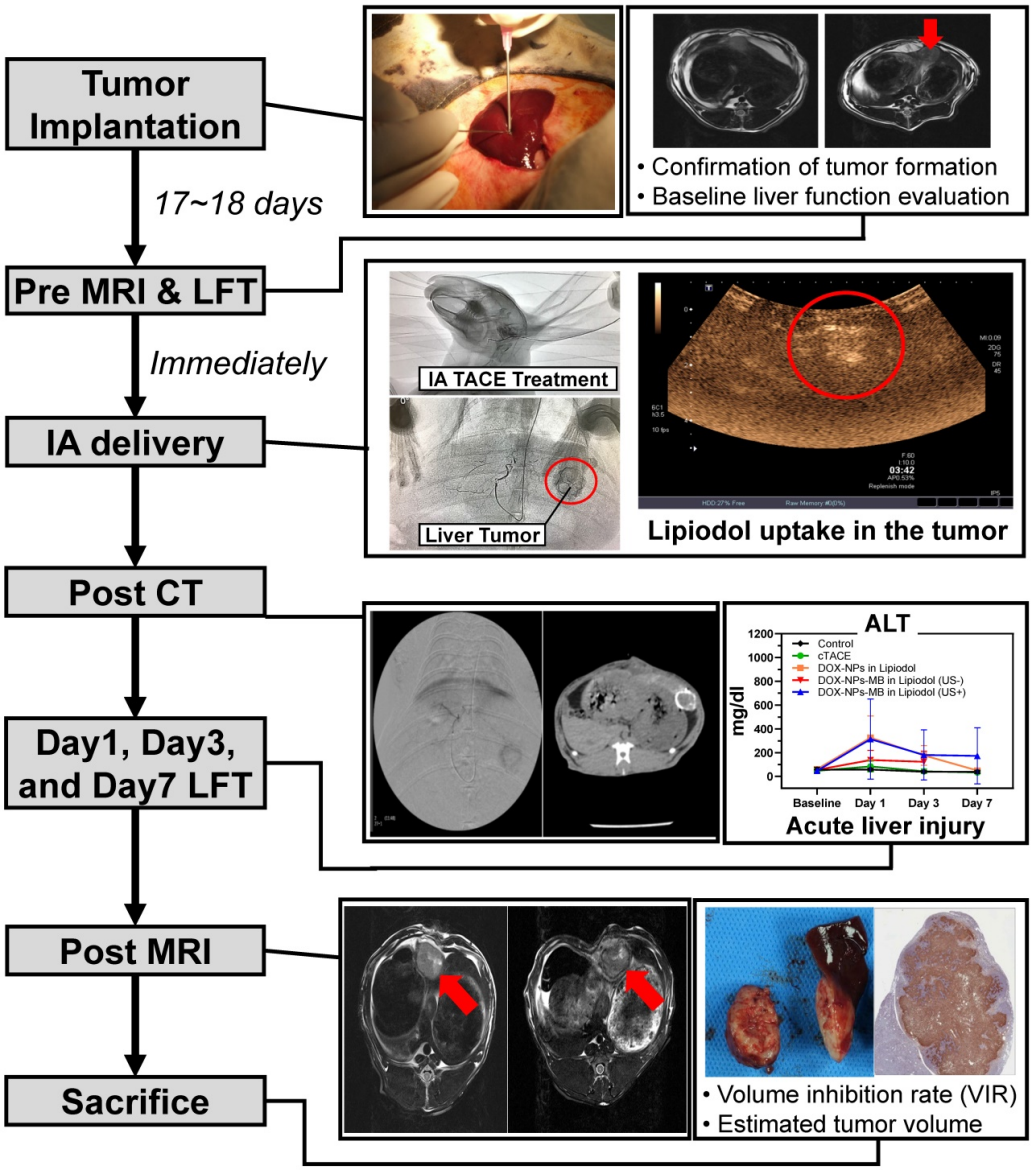
Experimental procedure with rabbits to verify liver cancer treatment efficacy and toxicity of DOX-NPs-MB complex in Lipiodol emulsion formulation, compared to the conventional TACE formulation.

**Figure 6:**
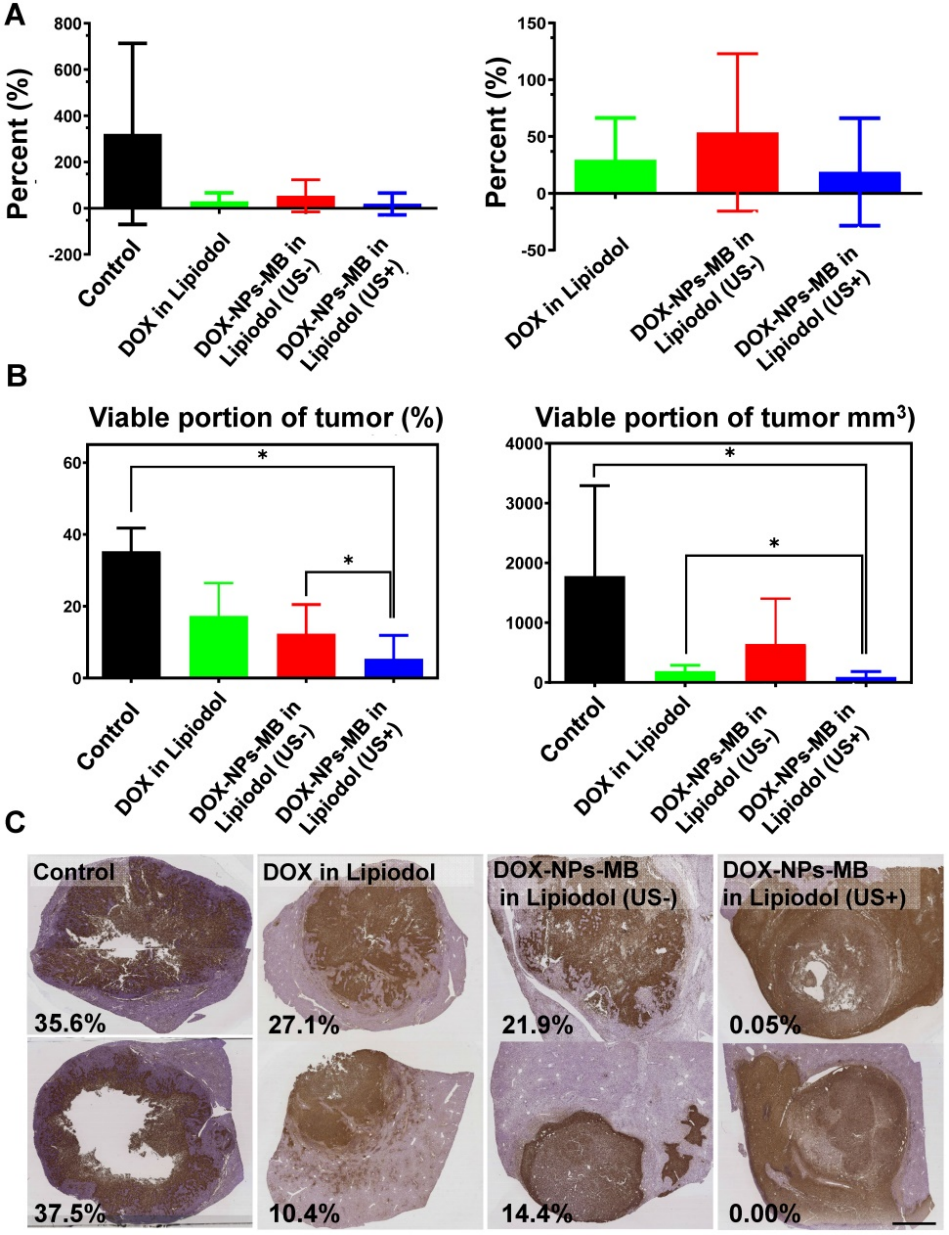
***In vivo* therapeutic efficacy of DOX-NPs-MB complex in Lipiodol compared with conventional TACE formulation, DOX in Lipiodol** (A) Comparison of tumor volume growth inhibition with each formulation. Right graph is the comparison of tumor volume excluding control group. (B) Analysis of the proportion of viable cancer cells in a tumor after cancer treatment with each formulation. The left graph demonstrates percentage of viable portion of tumor and the right indicates the viable tumor volume. (C) Representative histological segmentation of images obtained from each group, and quantitative analysis of viable tumor fraction. The scale bar indicates 5 mm.

**Figure 7:**
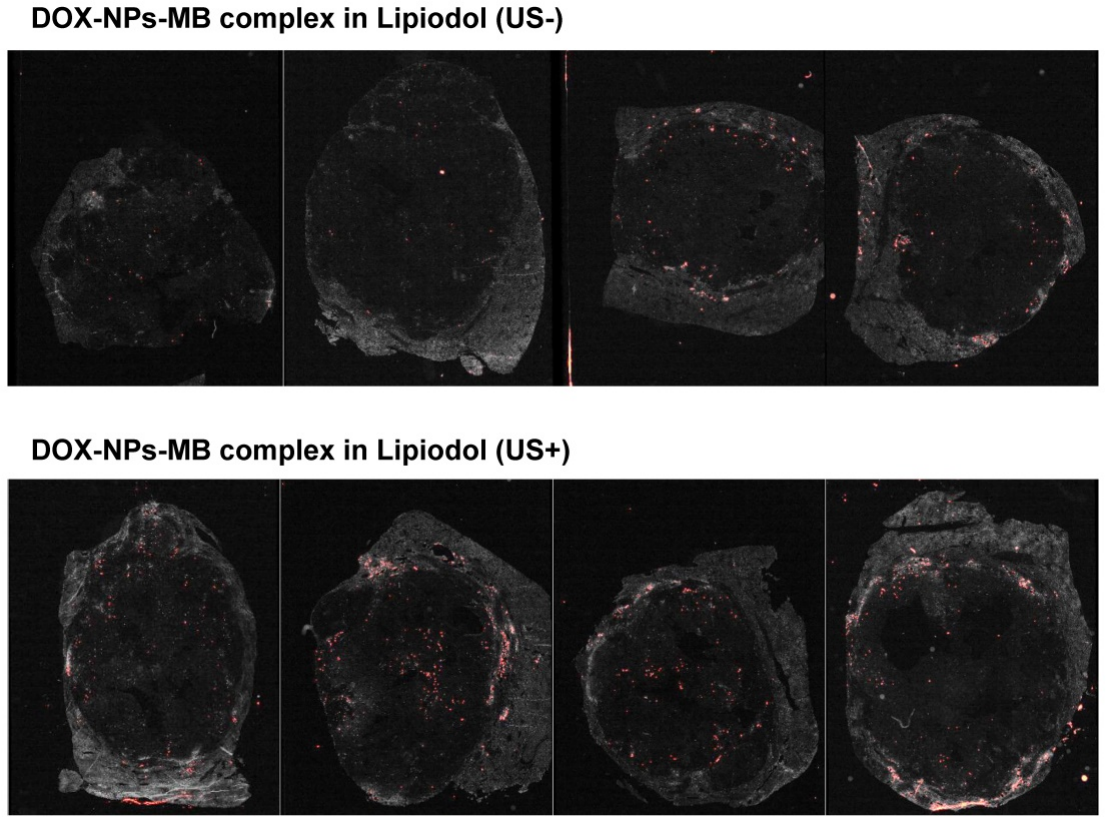
*In vivo* HCS experimental data showing the distribution of nanoparticles in liver tumor with or without ultrasound irradiation after infusion of DOX-NPs-MB complex in Lipiodol formulation. Red spots indicate the distribution of Alexa 647-labeded nanoparticles in the hepatic tumors.

The *in vivo* experiment was conducted as described in Figure 5. A 0.24 mL aliquot of emulsion was injected into the correct hepatic artery of the tumor-bearing rabbit. The final amount of doxorubicin delivered into the liver tumor of each rabbit was 0.5 mg, and therefore, the initial concentration was set to 6.25 mg/mL. All animals undergoing TACE were subjected to non-contrast computed tomography (CT) to confirm Lipiodol uptake in the tumor area (Figure S3). The anticancer effect of DOX-NPs-MB complex in Lipiodol emulsion was evaluated via quantitative MR and histopathologic analysis of viable tumors (Figure 6). Based on the MRI imaging analysis, three groups of animals treated with the drug, including DOX in Lipiodol, DOX-NPs-MB complex in Lipiodol (US-) and DOX-NPs-MB in Lipiodol (US+), showed significantly smaller tumor size than the control group (Figure 6A, left). The DOX-NPs-MB complex in Lipiodol (US+) group showed the smallest tumor size on average; however, the results did not show any statistically significant difference between the three groups (*p* > 0.05) (Figure 6A, right). Based on the histopathology of the viable tumors (Figure S4), the DOX-NPs-MB complex in Lipiodol (US+) group demonstrated the lowest viable fraction of the tumor in pathology studies, compared with the other groups (*p* < 0.05) (Figure 6B). There was no statistically significant difference in the extent of viable tumor between the group treated with DOX in Lipiodol and the group treated with DOX-NPs-MB complex in Lipiodol (US-) (*p* = 0.382). However, the group treated with DOX-NPs-MB complex in Lipiodol (US+) showed a significantly lower viable tumor compared with the group exposed to DOX in Lipiodol (*p* < 0.05). The measurements of viable tumor volume are presented in Figure 6B. The smallest viable tumor volume was detected in the group treated with DOX-NPs-MB complex in Lipiodol (US+) (*p* < 0.05). Differences in treatment effects between groups exposed to the DOX-NPs-MB complex in Lipiodol (US+) and the DOX-NPs-MB complex in Lipiodol (US-) indicate a cavitation effect of ultrasound treatment in the group treated with the DOX-NPs-MB complex in Lipiodol emulsion, which improved the permeability of the hepatic tumor vessels, and enhanced the delivery of doxorubicin-encapsulated nanoparticles to the hepatic tumor. The distribution of viable tumor cells in hepatic tumors by TUNEL assay of hepatic tumor fragments in each group is presented in Figure 6C. The groups treated with DOX in Lipiodol and DOX-NPs-MB complex in Lipiodol (US-) showed viable tumor cells in the peripheral and interstitial portions of the tumor, whereas DOX-NPs-MB complex in Lipiodol (US +)-treated group showed a consistent loss of tumor cell apoptosis, including the tumor periphery (Figure 6C). Cancer recurrence is caused by residual cancer cells in the peripheral region of the tumor. Therefore, the results of TUNEL assay (Figure S5) demonstrate the possibility of effective prevention of cancer recurrence, by delivering the chemotherapeutic agent using ultrasound irradiation and killing cancer cells in the peripheral region. In order to confirm the cavitation effect of microbubbles on the delivery of nanoparticles encapsulating chemotherapeutic agent into the hepatic tumor, the distribution of fluorescently conjugated nanoparticles with or without ultrasound irradiation was determined by HCS analysis (Figure 7). A relatively higher number of particles can be seen in the DOX-NPs-MB complex in Lipiodol (US+)-treated group, indicating the sonoporation effect of microbubbles on the delivery of nanoparticles into tumors.

### Hepatic toxicity

**Figure 8:**
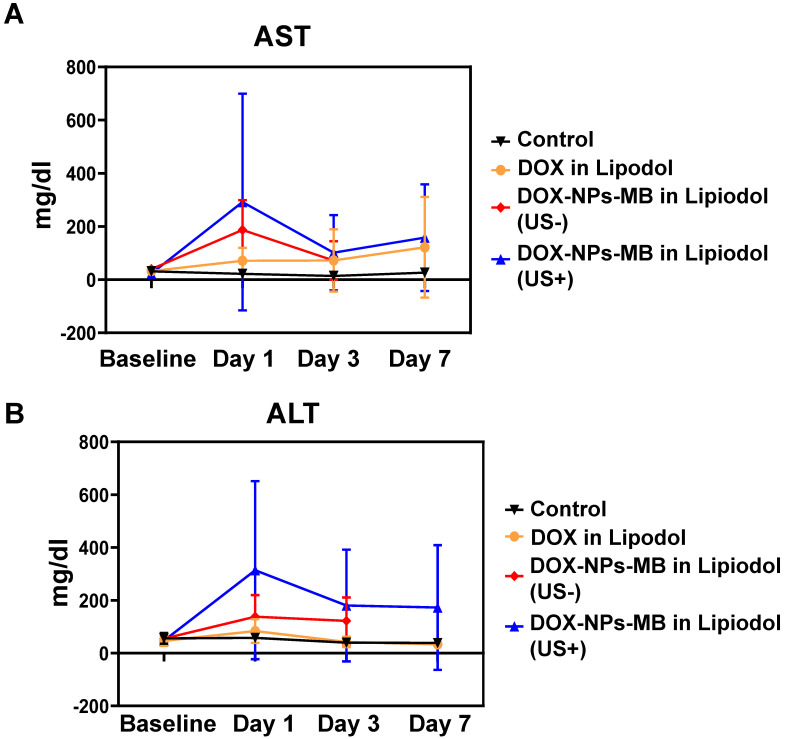
***Hepatic toxicity***(A) AST and (B) ALT values (for 7 days) after treatment with each formulation: DOX in Lipiodol, DOX-NPs-MB complex in Lipiodol (US-), DOX-NPs-MB complex in Lipiodol (US+). The highest enzyme levels were seen on day 1 in the group treated with DOX-NPs-MB complex in Lipiodol (US+).

All rabbits showed a tendency to reach the highest serum concentrations of AST and ALT at 24 h after treatment, which eventually declined and returned to baseline on day 7 post-treatment. The AST and ALT values measured at specific time intervals did not differ significantly between the treatment groups (Figure 8). When the liver is damaged or destroyed by chemotherapeutic agents, AST and ALT in the tissue are released into the bloodstream. Therefore, the levels reflect liver damage. The highest enzyme levels were seen on day 1 in the group treated with DOX-NPs-MB complex in Lipiodol (US+), indicating that the combination of DOX-NPs-MB complex and ultrasound irradiation enhanced the efficacy of anticancer drugs. This result may suggest that the DOX-NPs-MB complex in Lipiodol (US+)-treated group carried a relatively large number of nanoparticles entering the tissue, compared with the group not exposed to ultrasound.

## Discussion

In this study, the DOX-NPs-MB complex in Lipiodol emulsion was developed as a new ultrasound-triggered TACE formulation to overcome the poor delivery of drugs due to rapid elimination of the anticancer drug into the systemic circulation and the increased side effects 27. The DOX-NPs-MB complex in Lipiodol emulsion was monitored in real-time during the TACE procedure because microbubbles were stable in the emulsion and resonated under exposure to ultrasound at a low MI value of 0.06 (Figure 3). During intra-arterial infusion, the cavitation of microbubbles at a high MI value of 1.5, created jet streams and enhanced the permeability of doxorubicin-encapsulated nanoparticles into the tumors. Doxorubicin was expected to be released continuously from the nanoparticles upon delivery into the tumor 28. The sustained release of chemotherapeutic agents from nanoparticles has two positive effects. First, when drug-encapsulating nanoparticles are delivered to tumors, they release the drug for an extended period of time, resulting in long-term treatment effects. Second, when nanoparticles leak into systemic circulation, the exposure of normal tissues to high concentrations of chemotherapeutic agents can be prevented and side effects can be minimized. In conventional TACE, the success of the procedure can be only determined by Lipiodol uptake in the tumor detected on the CT images. In contrast, ultrasound and CT-guided multidimensional real-time monitoring were facilitated by the newly developed emulsion formulation, DOX-NPs-MB complex in Lipiodol (Figure 5). Multi-dimensional real-time monitoring enables accurate intra-procedural assessment of drug delivery, and the irradiation of ultrasound energy with high MI induces sonoporation effects that enhance the efficiency of chemotherapeutic nanoparticles delivered into tumors.

Since the emulsion size of DOX-NPs-MB complex in Lipiodol was larger than the diameter of micro-vessels, the newly formulated emulsion was detected in the blood vessels as layers (Lipiodol oil phase - DOX-NPs-MB complex aqueous phase - Lipiodol oil phase), and not as an emulsion (Figure 4). This finding suggests that the DOX-NPs-MB complex is located in the vicinity of the blood vessel wall and a sufficient sonoporation effect can be expected upon exposure to ultrasound. The DOX-NPs-MB complex in Lipiodol showed a cavitation effect following ultrasound exposure, at an MI value of 1.5 (Figure 3B). The cavitation of microbubbles generated a jet stream, temporarily causing stress in the surrounding cell membrane, which is known as inertial cavitation 29. It generates pores between the cells of the tumor vascular wall and delivers doxorubicin-encapsulated nanoparticles efficiently into the tumor (Figure 1). Overall, the anticancer efficacy of DOX-NPs-MB complex in Lipiodol emulsion formulation (US+) was greater than that of DOX-NPs-MB complex in Lipiodol (US-). The difference in chemotherapy effect with or without ultrasound irradiation can be attributed to cavitation of microbubbles (Figure 6).

The conventional TACE formulation contains an emulsion of aqueous droplets in an oil phase in which the aqueous phase containing anticancer agents and the ratio of the oil phase occur in a volume ratio of 1:4. However, it was not appropriate to adjust the initial doxorubicin concentration in the newly developed DOX-NPs-MB complex in Lipiodol emulsion because of the low encapsulation efficiency of chemotherapeutic agent in nanoparticles. Therefore, the DOX in Lipiodol and DOX-NPs-MB complex in Lipiodol formulations used in this study was prepared at an aqueous-to-oil phase ratio of 1:2 to deliver 0.50 mg of doxorubicin. The TACE formulation depends on oil-to-aqueous phase ratio, the number of three-way pumping methods used, and the properties of anticancer drugs 30, 31. The oil phase component was less than in the conventional 1:4 emulsion ratio. Nonetheless, it was confirmed that the emulsion formulation was prepared correctly (Figure 2B). Emulsions generated with oil-to-aqueous phase ratios of 2:1 are known to be less stable than those with a 4:1 ratio 32. Although the DOX-NPs-MB complex in Lipiodol was stable during the *in vivo* experiment, the long-term stability needs to be evaluated.

In the *in-vivo* therapeutic efficacy study, there was no statistically significant difference in tumor size inhibition between the group treated with the conventional TACE formulation and with the newly developed DOX-NPs-MB complex in Lipiodol formulation (Figure 6A). However, compared with other groups, the group treated with DOX-NPs-MB formulation (US+) showed dramatic tumoricidal effects, including the tumor periphery in histopathologic analysis (Figure 6C and Figure S3). The follow-up imaging was performed only on day 7 following the treatment, which might be insufficient to demonstrate treatment outcomes clearly. From a pathologic perspective, the main strategy to prevent the recurrence of cancer is to control the residual tumor cells in the peripheral region of tumor, where tumor cells are directly located beside the healthy liver tissue and have potential to penetrate or metastasize. Thus, based on the results of TUNEL assay, treatment with the DOX-NPs-MB complex emulsion plus ultrasound showed promising anti-cancer efficacy, in effectively suppressing cancer recurrence 33. The results of HCS analysis demonstrated the distribution of doxorubicin loaded nanoparticles (Figure 7). The nanoparticles were consistently located in the peripheral region of the tumor tissue where continuous release of doxorubicin elicited sufficient and sustained therapeutic effects.

## Conclusion

We developed a DOX-NPs-MB complex in Lipiodol emulsion formulation to overcome the disadvantages of conventional TACE formulations and to maximize treatment efficacy. Microbubbles, an ultrasound contrast agent used in the formulation, enabled real-time imaging of the TACE procedure with ultrasound, during the infusion of DOX-NPs-MB complex in Lipidol emulsion into the tumor-feeding vessels. In addition, the tumor was exposed to ultrasound of high mechanical index and the sonoporation effect induced by cavitation of microbubbles effectively delivered nanoparticles carrying chemotherapeutic agents into tumors, resulting in improved treatment efficacy compared to conventional TACE. The new concept of ultrasound sensitive TACE formulation described in this study represents a new treatment paradigm in liver cancer.

## Abbreviations

TACE): Transarterial chemoembolization
HSA: Human serum albumin
DOX-NPs: doxorubicin-loaded albumin nanoparticles
MB: microbubbles
2-IT: 2-iminothiolane hydrochloride
DSCP: 1,2-distearoyl-sn-glycero-3-phosphocholine
DSPE-PEG2k-NHS: 1,2-distearoyl-sn-glycero-3-phosphoethanolamine-N-[amino(Polyethylene Glycol)2000] (Ammonium Salt)
US: ultrasound
HPLC: high-performance liquid chromatography
PDMS: polydimethylsiloxane
DAPI: 4',6-diamidino-2-phenylindole
PFA: Paraformaldehyde
BABB: Benzyl Alcohol and Benzyl Benzoate
MR: Magnetic resonance
IVIM: Intravoxel incoherent motion
IA: intra-arterial
VIR: volume inhibition rate
ADC: value of the apparent diffusion coefficient
TUNEL: terminal deoxynucleotidyl transferase dUTP nick-end labeling
AST: aspartate transaminase
ALT: alanine transaminase
HCS: high-content screening
