# Supplementary material for: Development and evaluation of an ultrasound-triggered microbubble combined transarterial chemoembolization (TACE) formulation on rabbit VX2 liver cancer model
Source: Theranostics. 2021 Jan 1;11(1):79–92. doi: 10.7150/thno.45348 (PMC7681087; doi:10.7150/thno.45348)
Supplement: Supplementary file 1 — Supplementary figures. [file thnov11p0079s1.pdf]

Supplementary Information

Supplementary figure 1: Stability of DOX-NPs-MB complex in Lipiodol

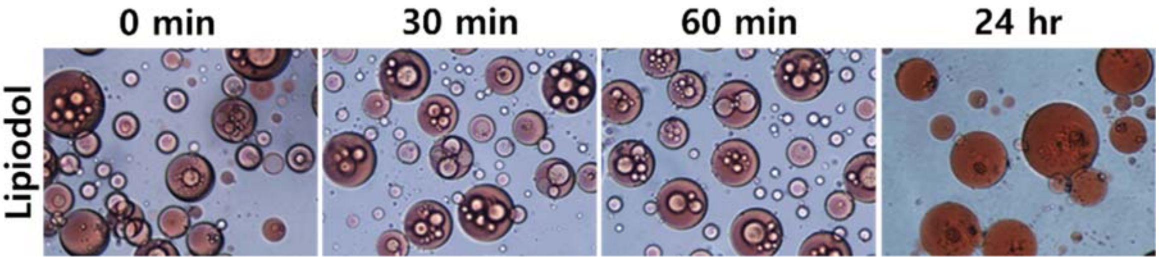

The DIC images of emulsion of DOX-NPs-MB complex in Lipiodol were obtained in different time points. In lipiodol solution, the size of DOX-NPs-MB emulsion remained almost same until 1hr.

Supplementary figure 2: Intravascular distribution of the emulsion of DOX-NPs-MB complex in Lipiodol in normal liver

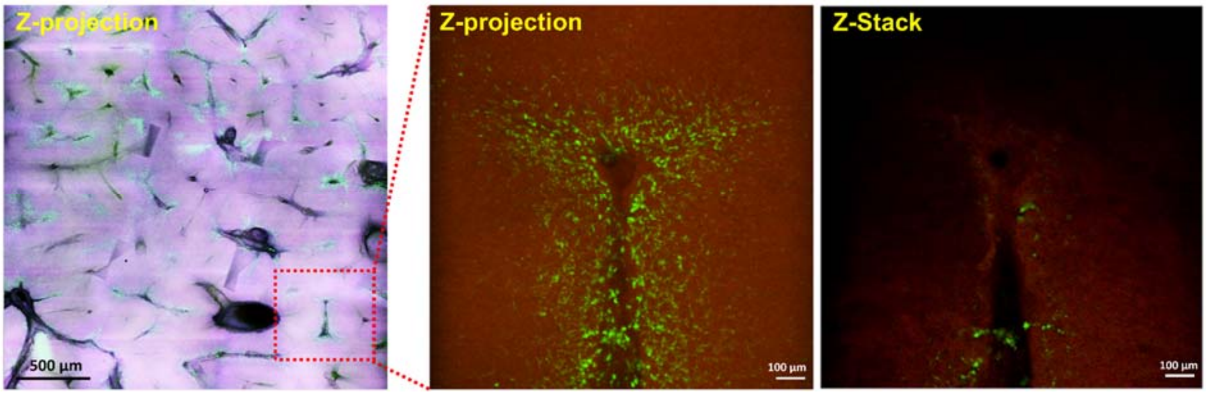

The distribution of the emulsion of DOX-NPs-MB complex in Lipiodol in normal tissue of the liver with VX2 tumor using a custom-built video-rate laser-scanning confocal microscopy system (IVMV imaging system).

Supplementary figure 3: Post-CT images of all group

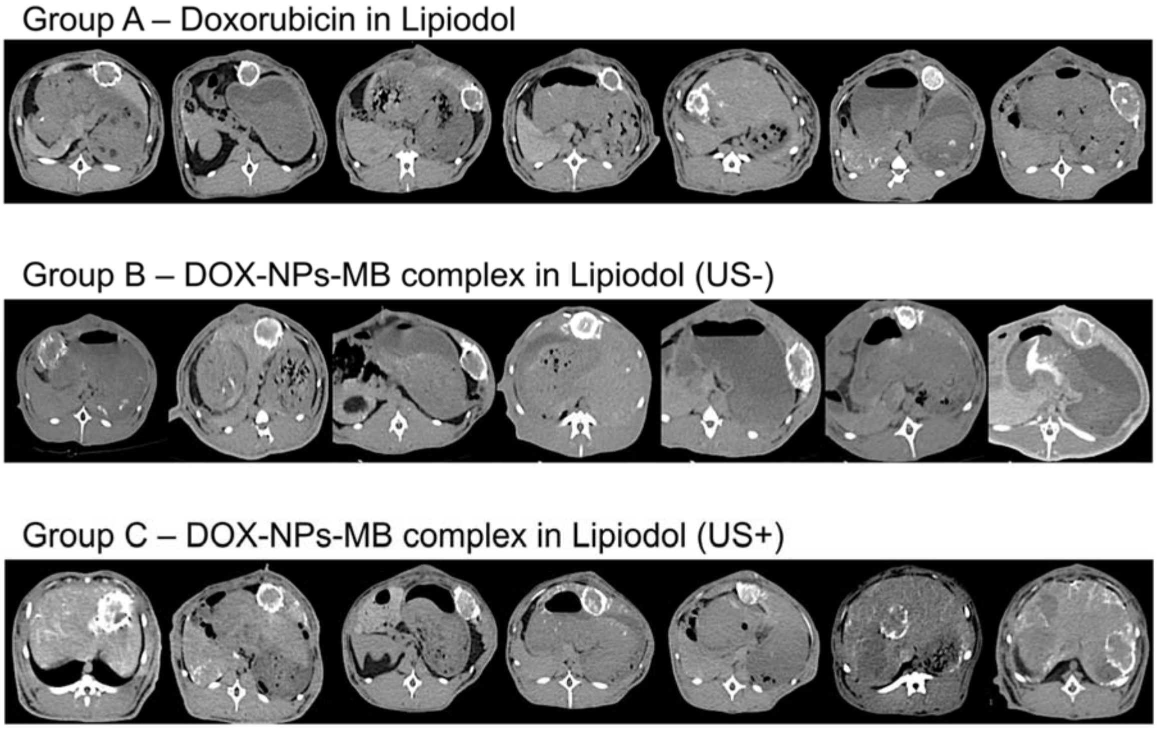

Noncontrast computed tomography (CT) scans were obtained from all animals which were treated by Lipiodol-containing emulsions, to confirm Lipiodol uptake in tumor areas successfully (Fig. 1). Immediately after infusion, rabbits were moved to CT room to be underwent CT scan. Under anesthesia with supine position, CT scan for abdominal areas were performed. Lipiodol deposition was clearly visible in all the tumor areas on the CT scans of all groups.

Supplementary figure 4: Histo-segmentation analysis of tumor section for viable tumor percentage calculation

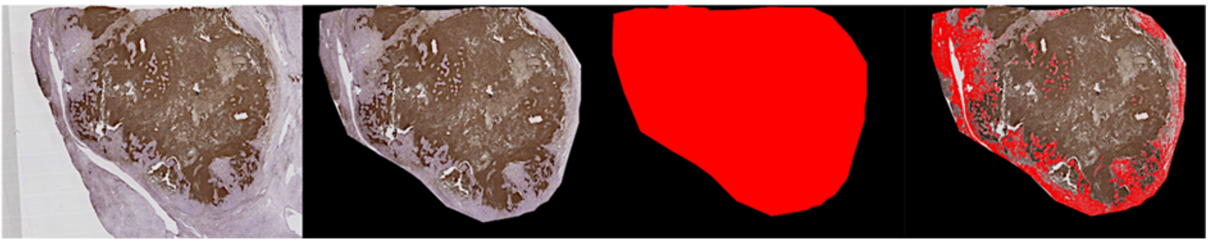

On gross inspection of the extracted tumor specimen cut in 1-mm thickness, a section of the largest tumor area was chosen and embedded in a paraffin block. The block was sectioned at a thickness of 5 μm for subsequent microscopic evaluation. Terminal deoxynucleotidyl transferase deoxyuridine triphosphate nick end labeling staining was performed to determine tumor necrosis and viability rates in all groups. All digitalized histopathologic images were evaluated using ImageJ software (National Institutes of Health, Bethesda, Maryland). One independent investigator who are blinded to the information regarding group of specimens draw whole tumor area first and evaluated viable portion of the tumor by free-hand drawing. The percentage area of viable tumors was calculated by dividing the pixel number representing the viable area by the total pixel number representing the whole tumor area. The estimated viable tumor volume was calculated by multiplying the tumor volume measured on day-7 MRI by percentage area of viable tumors.

Supplementary figure 5: Histosegmentation images of all group

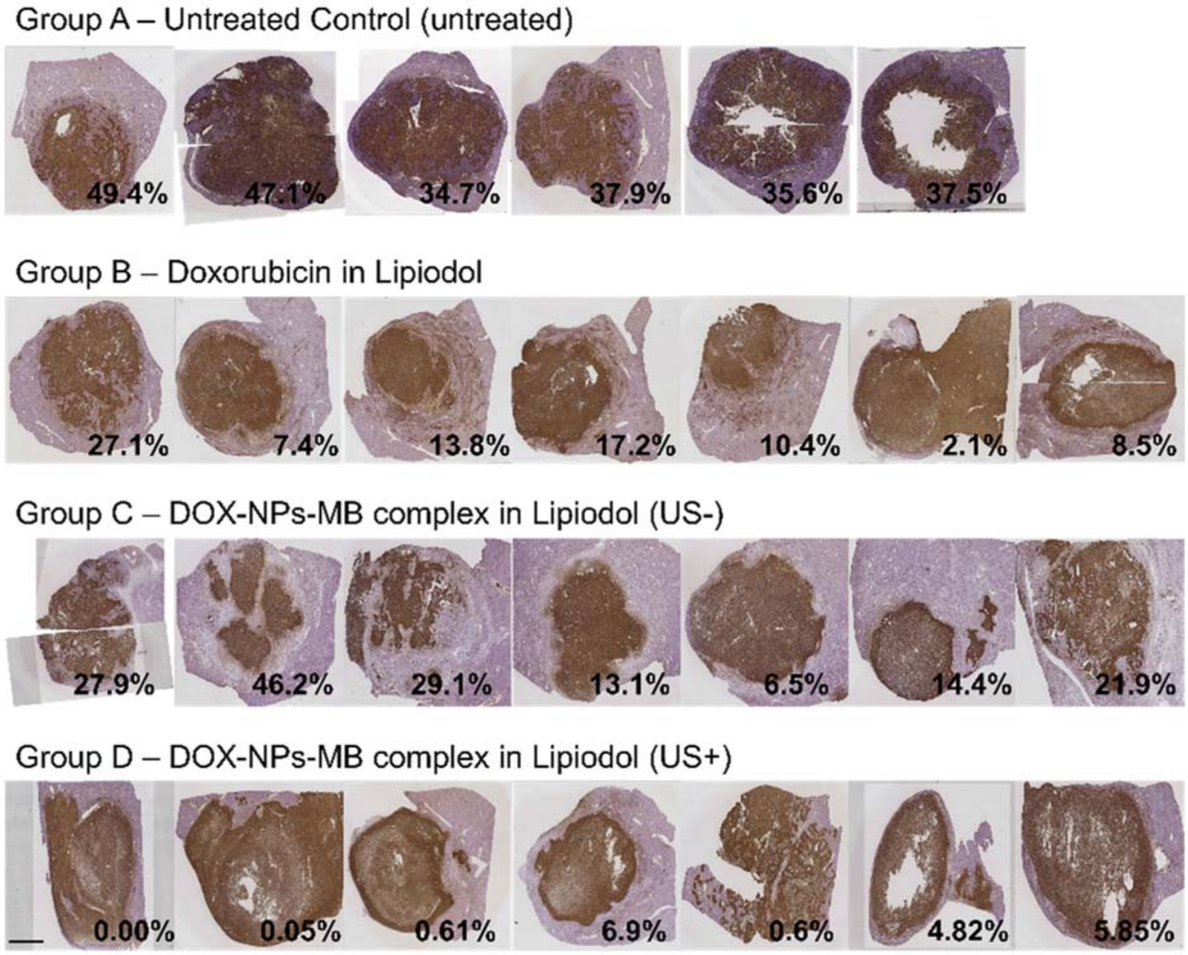

All tumors of each group were bisected and analyzed as above mentioned. Viable tumor percentage were calculated and presented in each image. The scale bar indicates 5 mm.
